# Efficient and tunable frequency conversion using periodically poled thin-film lithium tantalate nanowaveguides

**DOI:** 10.1515/nanoph-2025-0201

**Published:** 2025-08-05

**Authors:** Simin Yu, Mingyue Qi, Huizong Zhu, Bofu Zhao, Jingchun Qian, Yiqun Wu, Qiushi Chen, Juanjuan Lu

**Affiliations:** State Key Laboratory of Quantum Functional Materials, School of Information Science and Technology, 387433 ShanghaiTech University, Shanghai, 201210, China

**Keywords:** thin film lithium tantalate, periodic poling, second harmonic generation, integrated photonics

## Abstract

Thin-film lithium tantalate (TFLT) has recently emerged as a promising photonic platform for chip-scale nonlinear optics due to its weaker photorefraction, higher optical damage threshold, broader transparency window, and lower birefringence compared to that of thin-film lithium niobate. Here we report the first functional second harmonic generator achieved through high-fidelity poling of z-cut TFLT waveguides, based on a low-loss lithium tantalate integrated photonic platform. As a result, quasi-phase matching is performed between telecom (1,550 nm) and near-visible (775 nm) wavelengths in a straight waveguide and prompts strong second-harmonic generation with a normalized efficiency of 229 %/(W·cm^2^). An absolute conversion efficiency of 5.5 % is achieved with a pump power of 700 mW in the waveguide. Such a second-harmonic generator exhibits stable temperature tunability (−0.44 nm/°C), which is important for applications that require precise frequency alignment such as atomic clocks and quantum frequency conversion.

## 1 Introduction

The second-order nonlinearity (*χ* ^(2)^) is fundamental to many crucial nonlinear optical processes, including second-harmonic generation (SHG) [1], [2], sum frequency generation [3], and optical parametric oscillation [4], [5], [6]. Among these, SHG plays a particularly significant role in various applications, such as spectroscopy [7], [8], supercontinuum generation [9], [10], and quantum frequency conversion [11], [12]. Compared with other popular *χ* ^(2)^ materials like aluminum nitride [13], [14] and gallium arsenide [15], [16], ferroelectric materials including potassium titanyl phosphate [17], [18], lithium niobate (LN) [19], [20], and lithium tantalate (LT) [21], [22] stand out due to their large second-order nonlinear coefficients and flexibility in ferroelectric domain control. LN, in particular, has attracted considerable attention with the advent of thin-film LN (TFLN) technology, which has greatly advanced photonic integrated circuits, allowing the design of compact and high-performance optoelectronic chips [23], [24].

However, despite its popularity, TFLN has certain limitations like low optical damage threshold and strong photorefractive effect, restricting its performance under high power. Strategies such as material doping, improved crystal growth, and post-processing have been explored, but they also bring new challenges [25], [26]. As a result, the quest for novel thin-film ferroelectric materials has become a central focus of current research initiatives. The recent demonstration of high-quality thin-film LT (TFLT) has established it as an excellent alternative to TFLN. TFLT exhibits a comparable refractive index (*n* = 2.12 at 1,550 nm) and second-order nonlinearity (*d*_33_ = 26 pm/V at telecom wavelength) to TFLN [27], [28]. Moreover, TFLT demonstrates an enhanced optical damage threshold (240 MW/cm^2^ at 1,064 nm), a broader transparent window (0.28 − 5.5 μm), and a lower birefringence (0.004) further enhancing its potential for devices including electro-optic modulators, frequency converters, and optical switches [27], [29], [30]. Several SHG devices based on periodically poled LT (PPLT) on x-cut [22], [31] have already been developed, showcasing its promise for nonlinear photonic applications. Compared to x-cut, whose poling pattern can only be designed along the *z*-axis, the poling pattern of z-cut exhibits high flexibility, offering distinct advantages for on-chip integration [19], [32]. Furthermore, the poling process is simplified as it can be conducted directly in ambient air, eliminating the need for oil immersion or photoresist coating [31], [33].

In this paper, we present the first functional second-harmonic generator enabled by high-fidelity poling based on a low-loss z-cut TFLT photonic platform. As a result, quasi-phase matching (QPM) is realized between telecom (1,550 nm) and near-visible (775 nm) wavelengths in a straight waveguide, yielding strong SHG with a normalized efficiency of 229 %/(W·cm^2^). A maximum absolute conversion efficiency of 5.5 % is realized at a pump power of 700 mW in the waveguide. Notably, this second harmonic generator exhibits stable thermal tunability with a rate of −0.44 nm/°C, which is important for applications that require precise frequency alignment, such as atomic clocks and quantum frequency conversion.

## 2 Device design and fabrication

**Figure 1:**
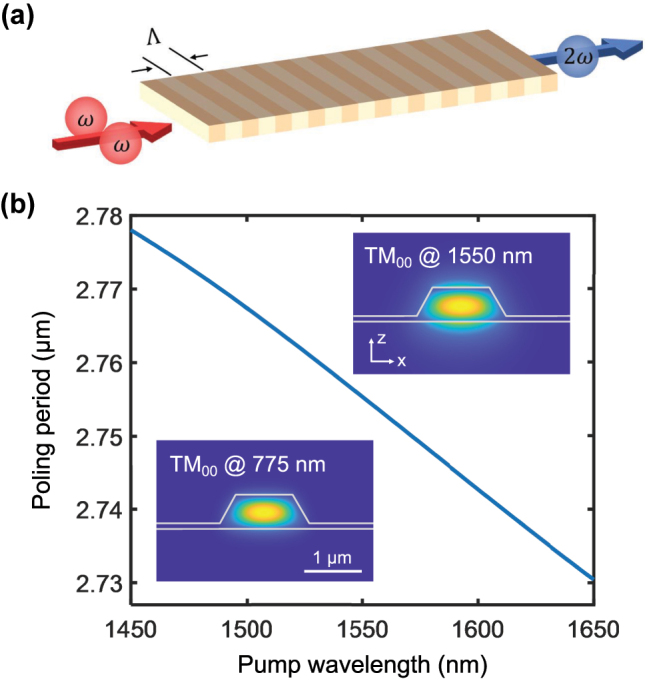
Design of the PPLT devices. (a) Schematic of SHG process in a PPLT waveguide, where the annihilation of two fundamental photons generates a second-harmonic photon. (b) Simulated poling period as a function of pump wavelength with a fixed film thickness of 600 nm and unetched layer of 100 nm, the refractive index is established in ref [34]. The insets show the simulated electric field distributions for the fundamental (top) and second harmonic (bottom) TM modes, respectively.

Figure 1a illustrates the design principle of the PPLT waveguide, where the SHG process produces a photon with twice the frequency by combining two photons of the fundamental frequency. The LT waveguide has a fixed width of 1 μm and a thickness of 600 nm with 100 nm thick unetched layer. The poling period Λ for QPM SHG at room temperature is determined by Λ = *λ*_2*ω*_/(*n*_2*ω*_ − *n*_*ω*_), where *λ*_2*ω*_ is the second-harmonic wavelength, while *n*_*ω*_ and *n*_2*ω*_ are the effective refractive indices at the first-harmonic (FH) and second-harmonic (SH) wavelengths, respectively. To utilize the highest second-order nonlinear tensor component *d*_33_, we simulate the poling period for the conversion of the fundamental transverse magnetic (TM_00_) mode from FH to SH wavelength, as shown in Figure 1b. The required poling period is estimated to be ∼2.75μm at a pump wavelength of 1,550 nm. The inserts show the numerically simulated optical mode profiles of TM_00_ mode at both FH (1,550 nm) and SH (775 nm) wavelengths.

**Figure 2:**
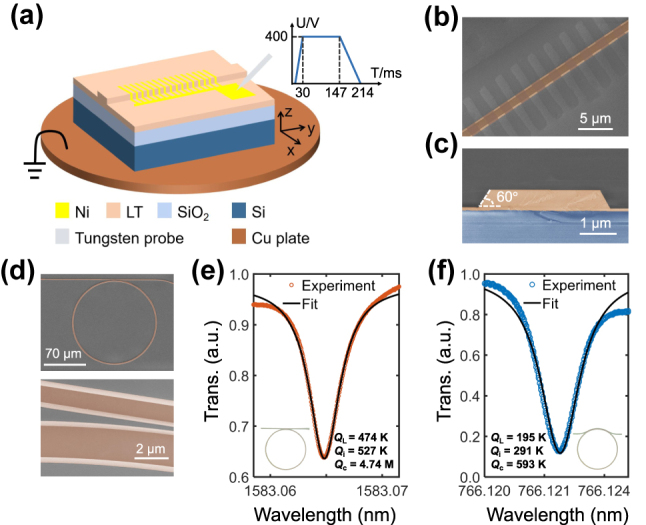
Device fabrication and propagation loss characterization. (a) Schematic diagram of the poling setup for z-cut LTOI devices. The insert shows the waveform of poling voltage pulse. (b–d) False-color SEM images of a PPLT waveguide (b), the cleaved waveguide facet with a sidewall angle of 60° (c), a microring with a radius of 70 μm and width of 1.8 μm, and its smooth sidewall in the coupling region (d), respectively. (e–f) Lorentzian fit of TM mode at FH wavelength (e) and SH wavelength (f) with extracted loaded (*Q*_L_), intrinsic (*Q*_i_), and coupling (*Q*_c_) *Q* values, measured from microrings coupled by the respective straight and pulley waveguides, as shown in the insets.

The device fabrication commences with the patterning of waveguides, followed by the poling process. A commercial lithium tantalate on insulator (LTOI) wafer (supplied by NanoLN) is utilized, consisting of a 600 nm-thick z-cut LT thin film on a 2.0 μm-thick silicon dioxide (SiO_2_) layer over a silicon substrate. The waveguide pattern is defined using electron beam lithography (EBL) with hydrogen silsesquioxane resist and developed in 25 % TMAH for high contrast. An optimized inductively coupled plasma reactive ion etching with Ar^+^ plasma transfers the pattern onto the LT layer. The chip is subsequently immersed in a solution of 3:1 KOH (40 %):H_2_O_2_ (30 %) for 3 h at 40 °C to remove the redeposition generated by dry etching. For the poling process, nickel (Ni) finger electrodes are first deposited on the LT waveguides through EBL and liftoff processes, aligned to the central 2.5-mm-long region of each LT waveguide. The chip is heated to 250 °C on a copper plate and then subjected to three 400 V, 120 ms pulses via a probe, as depicted in Figure 2a. The waveform of applied voltage pulse is shown in the inset. After removing Ni, the inverted domains are clearly visible by scanning electron microscope (SEM), exhibiting a duty cycle approaching 50 %, as illustrated in Figure 2b. The high poling fidelity is essential for achieving high conversion efficiency. The waveguide, with a width of 1 μm, is ultimately tapered to a width of 3 μm at both facets to improve the fiber-to-chip coupling efficiency. The total length of the waveguide is 6.5 mm, with a poling length of 2.5 mm. Figure 2c shows the cleaved waveguide facet with a sidewall angle of 60°. The insertion losses are calibrated to be −5.22 and −5.44 dB/facet for the telecom and near-visible wavelengths, respectively. The propagation losses of the TFLT waveguide are characterized by measuring the optical quality factor (*Q*) of the unpoled microring with a radius of 70 μm and width of 1.8 μm, as shown in Figure 2d. To achieve efficient coupling between microring resonator and bus waveguide, two distinct coupler designs are employed, tailored to their respective wavelength regimes. For telecom wavelength, a straight waveguide coupler with a width of 1.0 μm and a coupling gap of 0.9 μm is utilized. For near-visible wavelength, a pulley waveguide coupler is implemented, featuring a width of 0.63 μm and a coupling gap of 0.42 μm. A Lorentzian fit is, respectively, applied to the measured resonance dips of the TM mode transmission spectra around 1,583 nm and 766 nm, as shown in Figure 2e and f, yielding propagation losses of 0.72 dB/cm in the telecom wavelength and 2.69 dB/cm in the near-visible wavelength.

## 3 Results and discussion

**Figure 3:**
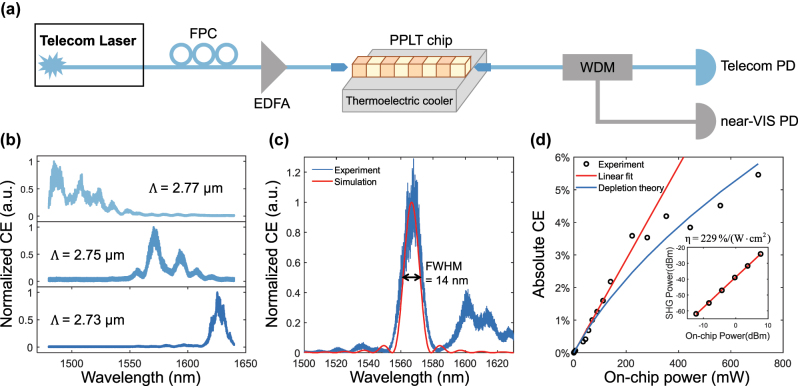
Second harmonic generation measurements. (a) Illustration of experimental setup for the characterization of the PPLT waveguides. FPC, fiber polarization controller; EDFA, erbium-doped fiber amplifier; WDM, wavelength division multiplexer; PD, photodetector. (b) SHG spectra of PPLT waveguides with varying poling periods at 25 °C. (c) Normalized SHG efficiency versus pump wavelength with Λ = 2.73 μm measured at 150 °C, indicating a FWHM of 14 nm and matching well with the numerical simulation. (d) Absolute conversion efficiency as a function of on-chip pump power. The inset presents the SHG-pump power relation in the non-depleted regime.

Figure 3a depicts the experimental setup for SHG measurement and device characterization. A tunable telecom laser (Santec TSL570) serves as the pump source, with a fiber polarization controller ensuring that the on-chip pump light is aligned to the TM polarization. The telecom and near-visible outputs are separated using a wavelength division multiplexer and subsequently measured by the corresponding photodetectors. The phase-matching wavelengths of devices, measured at 25 °C, for poling periods of 2.77, 2.75, and 2.73 μm, are found at 1,485, 1,571, and 1,626 nm, respectively, as shown in Figure 3b. These experimental results align well with the simulations presented in Figure 1b, confirming both the effectiveness and precision of the poling process. Figure 3c shows a typical *sinc* ^2^-like normalized conversion efficiency spectrum with a phase-matching wavelength of 1,568 nm, exhibiting a full width at half maximum (FWHM) of 14 nm. The slight deviation between the simulated (red line) and experimental data (blue line) is possibly attributed to the non-uniformity of poling and film thickness. To further increase the FWHM bandwidth for applications requiring broad spectral operation, aperiodic poling design and waveguide dispersion engineering could be employed.

The power dependence of the conversion efficiency has also been investigated through an erbium-doped fiber amplifier to amplify the optical power from the pump laser. The highest absolute conversion efficiency is measured to be 5.5 % at a pump power of 700 mW, as shown in Figure 3d. The experimental pump depletion behavior aligns well with the theoretical prediction  [35]. A linear fit in the non-depleted regime suggests a quadratic dependence of SHG power on pump power (inset of Figure 3d), resulting in an on-chip normalized SHG efficiency of 229 %/(W·cm^2^). We note that the recorded normalized efficiency is lower than the theoretical value of 5,142 %/(W·cm^2^), which is mainly attributed to the material defects (vacancy and inhomogeneity of the LT thin film) and poling imperfections (deviation of poling period and duty cycle). Further improvement could be envisioned with high-quality LTOI wafers and adapted control on the poling period [36]. Additionally, optimizing waveguide geometry and implementing post-fabrication annealing could help mitigate propagation losses and improve phase matching.

**Figure 4:**
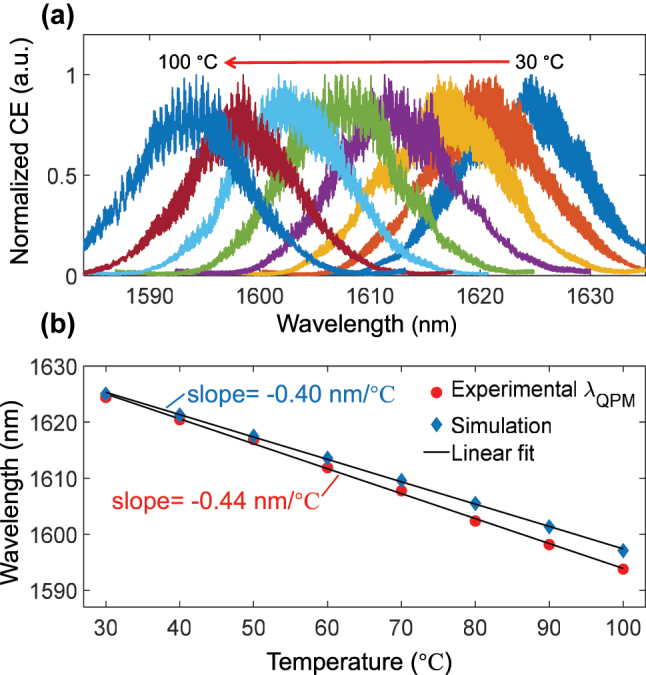
Temperature-dependent QPM characteristics. (a) Blue shift of the measured SHG spectra as the temperatures increased in increments of 10 °C from 30 °C to 100 °C. (b) The temperature dependence of QPM wavelength corresponding to (a), with the experimental and simulated tunability fitted to be −0.44 nm/°C and −0.40 nm/°C, respectively.

The thermal tunability of our QPM waveguide is systematically investigated. Figure 4a shows a distinct blue-shift of the SHG peak wavelength with the increasing temperature, exhibiting a thermal tunability of −0.44 nm/°C as implied in Figure 4b. This temperature-dependent behavior originates from two principal mechanisms: thermo-optic coefficient of the LT material and thermal expansion of poling period Λ. Following the established relationship for thermal expansion of the poling period  [37], and incorporating thermal-optical effect of LT material *n*_*e*_(*T*) established in  [38], our simulation predicts a tuning rate of −0.40 nm/°C, which agrees well with experimental measurements, as indicated in Figure 4b. The slight discrepancy between simulation and experiment can be possibly attributed to the approximation dn_*o*_/*dT* = 0 and fabrication-induced variations in waveguide dimensions. Nevertheless, the remarkable consistency between theory and experiment provides strong evidence for the reliability of thermal tuning characteristics of our device and confirms the effectiveness of our design approach for thermally tunable nonlinear photonic devices.

## 4 Conclusions

In conclusion, we have successfully demonstrated the first functional second harmonic generator based on high-fidelity poling based on a low-loss z-cut TFLT photonic platform. We achieve SHG with a normalized efficiency of 229 %/(W·cm^2^), and a maximum absolute conversion efficiency of 5.5 % at a pump power of 700 mW in the waveguide. The demonstrated temperature tunability of −0.44 nm/°C further reinforces the potential for precise frequency alignment, which is critical for many precision applications. Our work not only offers significant insights into optimizing SHG performance, but also establishes a promising foundation for future applications in fields such as quantum frequency conversion and atomic clocks. Moreover, the performance can be further optimized by reducing propagation losses and improving poling quality, paving the way for even more impressive applications of second harmonic generator on the TFLT platform.
